# Leukocytosis and Spurious Hypoxemia

**DOI:** 10.7759/cureus.15942

**Published:** 2021-06-26

**Authors:** Sachin Gupta, Sushma Medikayala, Balraj Singh, Harshil Bhatt, Sandeep Singh

**Affiliations:** 1 Hospital Medicine, Tower Health Reading Hospital, West Reading, USA; 2 Nephrology, Cleveland Clinic Florida, Weston, USA; 3 Hematology/Oncology, Saint Joseph's University Medical Center, Paterson, USA; 4 Internal Medicine, Goshen Hospital, Goshen, USA; 5 Internal Medicine, Indiana University School of Medicine, South Bend, USA

**Keywords:** spurious hypoxemia, oxygen steal, leucocyte larcency, aml, leucocytosis and abnormal abg

## Abstract

Abnormally low pO2 and oxygen saturations on arterial blood gases (ABGs) test have been reported in the patients who have very high WBC and platelet counts; generally in the setting of hematological malignancies. This is presumably related to the consumption of oxygen by the active cellular elements in the arterial blood sample during the process of ABG analysis. This phenomenon which is also known as "spurious hypoxemia" or "oxygen steal" or "leukocyte/platelet larceny" is suspected when there is no other obvious explanation for hypoxemia on ABG, especially in the setting of normal oxygen saturations by the pulse oximetry. It is important for medical professionals to be aware of this condition so that appropriate workup and triage can be performed on such patients, which may otherwise lead to unnecessary hospitalization and escalation of care.

## Introduction

The phenomenon of "spurious hypoxemia" has been described in patients with very high WBC and platelet counts especially in patients with leukemia or myeloproliferative disorders [1]. Also known as "leukocyte/platelet larceny" or "oxygen steal" is related to the in vitro consumption of the oxygen by active leukocytes and platelets in the arterial blood sample manifesting as low pO2 and thus low oxygen saturations on the ABGs [2]. At the same time, continuous pulse oximetry may show oxygen saturations within normal limits [2]. Timely recognition of spurious hypoxemia is important because it helps to avoid misdiagnosis, unnecessary radiological/blood work, cost, and effort of overtreatment. Here we present an interesting case of “spurious hypoxemia” in a 93-year-old woman with a history of acute myeloid leukemia (AML) along with the review of relevant literature.

## Case presentation

A 93-year-old female with a past medical history of AML, anemia, and thrombocytopenia (secondary to leukemia) was admitted to the hospital for elective packed red blood transfusions. Initial labs showed a high WBC count of 81000/mm3 (blasts 56%, lymphocytes 14%, monocytes 20%, and segmented neutrophils 10%), hemoglobin, and hematocrit of 6.4 g/dl and 17%, and platelet count of 7000/ mm3.

On examination, the patient appeared comfortable with no respiratory distress. Physical examination was unremarkable other than significant pallor in conjunctiva. The patient had a good follow-up with the medical oncologist and received a single cycle of chemotherapy with decitabine in the past, along with multiple blood transfusions for severe anemia and thrombocytopenia. The patient's ABG was drawn later in the day mistakenly as it was ordered for another patient. Surprisingly, the ABG on room air showed significant hypoxemia with a pH of 7.48, pCO2 35 mm Hg, pO2 45 mm Hg, bicarbonate 26.1, and oxygen saturation of 84%. An urgent ICU consult was requested for abnormal ABG results. It was found that the patient was having oxygen saturation of 98-99% on room air on the pulse oximeter. The patient's oxygen saturation by pulse oximeter remained unchanged after applying the probe on different digits and even with a forehead probe. The patient remained very comfortable throughout the course of the ICU evaluation. A chest X-ray was obtained which was grossly unremarkable with no evidence of infiltrates, pulmonary edema, atelectasis, pleural effusion or pneumothorax, etc. Also, her 2D echo which was performed a few months ago showed a normal ejection fraction of 60% with no evidence of diastolic dysfunction or valvular abnormalities. Thus, no obvious etiology for hypoxemia on ABG was evident at that point.

A suspicion for hypoxemia related to hyper-leukocytosis was considered and ABG was repeated with the syringe immersed in ice and taken to the lab immediately with some improvement in the parameters; raising the pO2 to 57 and saturation to 90%. On visual inspection, the arterial blood was bright red in color strongly suggesting an arterial sample. Though efforts were made to obtain ABG in a cyanide-containing syringe or to get a bedside ABG analyzer, we were unable to do so because of the unavailability of the above two items in the hospital. However, taking all the evidence into consideration, it was determined that the patient's hypoxemic picture on ABG was related to her very high leukocyte count and it was decided to follow up with the patient closely with continuous pulse oximetry and to watch for any deterioration in the respiratory status rather than doing aggressive workup or treatment for hypoxia. The patient was discharged home in stable condition the next day. She continued her follow-up with the primary oncologist for the next few weeks and was ultimately transitioned to home hospice as the patient declined chemotherapy and active treatment for her leukemia.

## Discussion

Various terms like "spurious hypoxemia", "leukocyte/platelet larceny", "oxygen steal" have been used for the phenomenon of a rapid drop in oxygen saturation on the blood gas analysis of the patients with very high WBC or platelet counts. This was first described by Hess et al. in 1979 [2]. It is important for the clinicians to be aware of this phenomenon as patients with leukemias, in general, are prone to have pulmonary compromise and hypoxemia because of several reasons like infections, intra-alveolar hemorrhage, pulmonary embolism, etc. It becomes crucial to be able to differentiate "true hypoxemia" from "spurious hypoxemia" as an inability to do so may lead to unnecessary investigations and treatments bringing concerns regarding cost and patient safety. There have been reported cases where patients were intubated and put on mechanical ventilation because of the inability to identify spurious hypoxemia [3].

It has been postulated that a high number of cellular elements in the blood specimen consume oxygen dissolved in the plasma in a rapid manner leading to an artifactual decline in pO2 and thus the calculated oxygen saturation from the ABG specimen [4]. On the other hand, pulse oximeter readings remain within normal limits or are unaffected. Thus, spurious hypoxia is suspected when there is a mismatch between the oxygen saturation on ABG compared to continuous pulse oximeter readings in a patient with the background of hyperleukocytosis or thrombocytosis. It has been found to be associated more with an increase in the number of cellular elements like immature WBCs, platelets, and reticulocytes than with others like mature RBCs or mature WBCs [2]. It has been postulated that immature cellular elements, especially leukemic cells and more specifically, blast form of granulocytes, have the highest metabolic rate, and consume oxygen at a faster rate compared to mature cellular elements [5, 6]. Mature RBCs are known to have an anaerobic metabolism and thus, do not consume oxygen.

Though classically described in the setting of acute leukemias, it has been reported in association with chronic leukemias as well [7,8,9]. Mizock et al. have shown a favorable role of continuous blood gas analysis in evaluating patients with spurious hypoxemia [10]. Studies have been completed to determine the effect of rapidly immersing the specimen in ice to decrease the metabolic rate and thus preventing the fall in oxygen saturation. Experiments performed by Wong et al. [9] and Chillar et al. [11] failed to show an appreciable effect of placing the specimen in ice water. Adding potassium cyanide to the blood specimen to inhibit the respiratory cycle of the WBCs has been evaluated and may be theoretically promising, but not always be practically possible [4]. Therefore, rapid processing of the blood specimen using bedside blood gas analysis or careful monitoring by pulse oximetry may be useful in the evaluation of the patients with suspected "spurious hypoxemia" related to very high blood cell counts.

## Conclusions

A very careful interpretation of the ABG is required in patients with hyperleukocytosis and thrombocytosis. Unexpected and extremely low blood oxygen saturation on ABG in the presence of normal oxygen saturation on pulse oximetry may be a spurious phenomenon and overall clinical and laboratory parameters should be evaluated before proceeding to further investigations and treatment.
